# Bivalent RSVpreF Subunit Vaccine Safety and Immunogenicity in Seropositive 2–<18 Year Olds

**DOI:** 10.3390/vaccines14020128

**Published:** 2026-01-28

**Authors:** Julia Glanternik, Grant C. Paulsen, Shelly Senders, Michael Smith, Emma Shittu, Barbara A. Pahud, Lisa Pereira, Lesong Chen, Maria Maddalena Lino, Elena V. Kalinina, Danielle Baranova, Warren V. Kalina, Elie Needle, MaryAnn Murillo, John M. Leech, David Cooper, Kena A. Swanson, Annaliesa S. Anderson, Alejandra Gurtman, Iona Munjal

**Affiliations:** 1 Pfizer Vaccines, Pfizer Inc., Pearl River, NY 10965, USA; 2 Department of Pediatrics, University of Cincinnati College of Medicine and Division of Pediatric Infectious Diseases, Cincinnati Children’s Hospital Medical Center, Cincinnati, OH 45229, USA; grant.paulsen@cchmc.org; 3 Senders Pediatrics, South Euclid, OH 44121, USA; 4 Division of Infectious Diseases, Duke University, Durham, NC 27710, USA; michael.j.smith@duke.edu; 5 Pfizer Vaccines, Pfizer Ltd., Marlow SL7 1YL, UK; 6 Pfizer Vaccines, Pfizer Inc., Cincinnati, OH 45202, USA; 7 Worldwide Safety, Pfizer Srl., 20152 Milan, Italy

**Keywords:** clinical trial, immunogenicity, pediatric, LRTI, RSV, RSVpreF, safety

## Abstract

**Background/Objectives:** We aimed to determine safe and immunogenic RSVpreF vaccine dose levels for further clinical development in 2–<18 year olds. **Methods:** The phase 1, age-descending, open-label Picasso trial evaluated different RSVpreF dose levels in respiratory syncytial virus (RSV)-seropositive 2–<5 year olds and 5–<18 year olds who were either healthy or had chronic medical conditions with increased RSV illness risk. Participants received a single dose of RSVpreF (60 µg or 120 µg dose level). The primary objective was to describe safety and tolerability at each dose level and age group, including frequencies of reactogenicity and adverse events (AEs). The secondary objective was to describe RSV neutralizing antibody responses at each dose level and age group 1 month after vaccination. **Results:** Overall, 127 participants received RSVpreF 60 µg (2–<5 year olds, *n* = 20; 5–<18 year olds, *n* = 35) or 120 µg (*n* = 24 and *n* = 48, respectively); 54% were male and 69% were White. Local reactions and systemic events were reported in 17–20% and 33–45% of 2–<5 year olds, respectively, and 49–56% and 52–60% of 5–<18 year olds; most were mild or moderate in severity. AEs were reported in 13–15% of 2–<5 year olds and 8–14% of 5–<18 year olds. No AEs leading to withdrawal or vaccine-related serious AEs were reported. RSV-A and RSV-B neutralizing titer geometric mean fold rises from before to 1 month after vaccination with RSVpreF 60 and 120 µg, which were 17.7–20.6 and 42.8–39.8, respectively, in 2–<5 year olds, and 19.0–23.5 and 20.3–20.3, respectively, in 5–<18 year olds. **Conclusions:** RSVpreF was safe, well tolerated, and elicited immune responses in RSV-seropositive 2–<18-year-old participants, supporting further clinical development in this pediatric population, including those with chronic conditions.

## 1. Introduction

Respiratory syncytial virus (RSV) infection is widespread, infecting nearly all children before their second birthday [1,2]. Most pediatric RSV infections are mild; however, some develop into RSV-associated lower respiratory tract illness (RSV-LRTI), such as bronchiolitis or pneumonia, which can lead to severe respiratory distress or even death [3,4,5]. Additionally, RSV may be associated with long-term sequelae, including asthma [1,4]. Among pediatric populations, risk factors for severe RSV-LRTI are often associated with immature or compromised immune and pulmonary systems and include age <6 months, premature birth, chronic respiratory disease or lung malformation, congenital heart disease, neuromuscular disease, cerebral palsy, cystic fibrosis, and Down syndrome [4,5,6,7,8,9,10].

Clinical management of severe RSV-LRTI is mainly supportive, and more prophylactic approaches are needed to protect children at high risk [11]. Maternal vaccination is available to protect infants against RSV-LRTI through 6 months of age [12]. Three monoclonal antibody (mAb) prophylaxes specific to the RSV F viral surface protein (i.e., palivizumab, nirsevimab, and clesrovimab) are also available currently for RSV-LRTI prevention in infants and children who remain vulnerable in their second RSV season [13,14,15].

The F protein on the virion exists in a metastable prefusion state, transitioning to a stable postfusion state while mediating virus entry into cells. The most potent neutralizing anti-RSV antibodies are specific to the prefusion form [16]. Costs and logistics required to implement widespread mAb use may be limited in certain settings, particularly in resource-limited countries where RSV-associated mortality is highest and no mAb is approved for protection of children against severe RSV-LRTI beyond their second RSV season [13,14,15,17].

In the 1960s, a formalin-inactivated whole virus RSV vaccine (FI-RSV) displaying the F protein in a postfusion conformation administered to RSV-naive children resulted in substantial vaccine-associated enhanced RSV respiratory disease (VAERD) upon subsequent natural infection, which clinically presented with fever, wheezing, and bronchopneumonia resulting in substantial morbidity [18,19,20,21]. Elucidation of the pathogenesis of VAERD identified immunization with vaccine antigens processed outside of the cytoplasm, resulting in immune signatures that included a predominantly non-neutralizing antibody response and T-helper type 2 (Th2)-dominant CD4+ T-cell response resulting in pulmonary deposition of eosinophils and immune complexes following natural RSV infection [18,21]. Throughout RSV vaccine development over the last several decades, VAERD has not been observed in individuals who were previously immunologically primed by natural RSV infection and who are RSV-seropositive, which includes most individuals 2 years and older [18].

Identifying the RSV prefusion F protein conformation as the primary target of the most potent RSV-neutralizing antibodies led to successful development of RSV vaccines, three of which are licensed in adult populations [12,17,22,23,24]. One of these, the bivalent RSV prefusion protein F (RSVpreF) vaccine, consists of stabilized prefusion F protein representing RSV-A and RSV-B subgroups [12,25]. RSVpreF elicits a high ratio of neutralizing responses to prefusion F-binding immunoglobulin G (IgG), thereby decreasing the likelihood of VAERD, which was characterized by a contrasting Th2-dominant response [18,26,27]. In phase 3 clinical studies, RSVpreF at the 120 µg dose level was efficacious in preventing RSV-LRTI in older adults [28] and RSV-associated severe LRTI in infants through 6 months of age through maternal vaccination [29]. It also elicited robust neutralizing responses and met immunobridging effectiveness criteria in individuals ≥18 years of age at high risk of RSV-LRTI [30]. Currently RSVpreF vaccination is licensed for use in adults 60 years and older, adults 18–<60 years of age at high risk for RSV-LRTI, and for use in pregnancy to protect infants through 6 months of age; no RSV vaccine is licensed for direct immunization of pediatric populations [12].

RSV is associated with substantial morbidity and mortality in at-risk populations, including young children and pediatric populations with underlying conditions for which there is no effective treatment or prophylaxes beyond supportive care [4,5,6,11,13,14,17]. Therefore, an unmet need remains for effective vaccines offering direct protection for at-risk pediatric populations.

Here, we present the first safety and immunogenicity results for direct vaccination with RSVpreF in pediatric populations from the completed phase 1 Picasso trial investigating RSVpreF two dose levels in RSV-seropositive children 2–<18 years of age.

## 2. Methods

### 2.1. Design

The phase 1, open-label Picasso study (ClinicalTrials.gov NCT05900154; registration date: 2 June 2023)*,* which was conducted at 16 US sites between 22 June 2023 and 29 February 2024, evaluated two different RSVpreF dose levels in 2–<18 year olds to identify dose level(s) for further clinical development in this age group. Participants were stratified into two age cohorts (i.e., 2–<5 and 5–<18 years) based on the rationale that preschool-aged children are likely to have fewer prior RSV exposures, lower baseline antibody titers, and smaller airway dimensions, whereas school-aged children 5 years and older are expected to have experienced more RSV infections, possess higher baseline titers, and exhibit larger airway anatomy. To assess the appropriate RSVpreF dose level for vaccination of the two pediatric age groups, an age-descending trial design was implemented. This design, often employed in vaccine development and recommended by regulatory agencies, involves sequential enrollment of progressively younger participants after safety, tolerability, and immunogenicity and/or efficacy have been characterized in older populations [31,32,33]. The age-descending design leverages comprehensive adult data to minimize risk and to ensure dose-level selection for pediatric populations is informed by prior evidence. Consistent with the design of early adult clinical trials, the dose levels selected were 120 µg (the licensed adult dose [12]) and 60 µg (which was administered to the younger age group before the 120 µg dose level).

Participants were healthy or had chronic medical conditions conferring increased RSV-LRTI risk (i.e., cystic fibrosis, medically treated asthma, chronic respiratory diseases or lung malformations, Down syndrome, neuromuscular disease, cerebral palsy, congenital heart disease). Only seropositive children could enroll. Serostatus testing of 2–<5 year olds was required to confirm RSV seropositivity (point-of-care serostatus test against non-vaccine antigens (RSV Ga, Gb, M, and/or N)) before enrollment; 5–<18 year olds were assumed to be RSV-seropositive based on their age. Further details on participant recruitment, eligibility criteria, and serostatus testing are provided in the Supplementary Text S1.

Study conduct complied with the protocol and consensus ethical principles derived from international guidelines (Declaration of Helsinki; Council for International Organizations of Medical Sciences), applicable International Council for Harmonisation Good Clinical Practice guidelines, and other relevant laws/regulations. All participants and their parents/guardians provided written informed assent (if able) and consent, respectively, before enrollment.

### 2.2. Intervention

Participants were assigned to the study intervention using interactive response technology. Participants were divided into two age groups (2–<5 and 5–<18 years). RSVpreF at the 120 µg dose level in a 0.5 mL volume was administered by study staff at the investigator site into the deltoid muscle in 5–<18 year olds first. Upon confirmation of an acceptable RSVpreF safety and tolerability profile by an internal review committee (IRC), RSVpreF vaccination at the 60 µg dose level in a 0.25 mL volume proceeded in 2–<5 year olds. Following IRC review and determination of an acceptable safety and tolerability profile for RSVpreF 60 µg in 2–<5 year olds, administration of RSVpreF 120 µg in that age group could proceed. RSVpreF 60 µg was also tested in 5–<18 year olds. Participants, caregivers, site personnel, and the sponsor were unblinded to assigned intervention.

### 2.3. Objectives, Endpoints, and Assays

The primary objective was to describe RSVpreF safety and tolerability at each dose level in 2–<5 year olds and 5–<18 year olds. Reactogenicity events were recorded in an electronic diary by the participant or parent/guardian for 7 days after vaccination (see Table S1 for the age-specific reactogenicity grading scale). Adverse events (AEs) were recorded through 1 month after vaccination, and serious AEs (SAEs), newly diagnosed chronic medical conditions (NDCMCs), and AEs of special interest (AESIs) were recorded through study completion. AESIs as collected in previous RSVpreF studies in adult populations included Guillain-Barré syndrome, acute polyneuropathy without an underlying etiology, atrial fibrillation, and hypertensive disorder of pregnancy diagnoses, as well as preterm delivery (i.e., <37 weeks’ gestation).

Blood samples for immunogenicity serological assessments and peripheral blood mononuclear cell (PBMC) isolation for T-cell response analyses were attained before vaccination and 1 month after vaccination. Secondary endpoints included RSV-A and RSV-B neutralizing geometric mean titers (GMTs) before and 1 month after vaccination and geometric mean fold rises (GMFRs) from before to 1 month after vaccination determined using immunologic assays described previously [26]. RSV neutralizing activity was determined by separately mixing serial dilutions of heat-inactivated sera with the RSV-A or RSV-B strains before transferring the mixture onto tissue culture plates containing an epithelial cell monolayer. After culturing for approximately 24 h, murine anti-RSV fusion glycoprotein mAb and an anti-murine secondary antibody stain were used to detect and quantitate viral foci. The neutralization titer was calculated as the reciprocal of the serum dilution that, by interpolation, corresponded to a 50% reduction in viral focus-forming units relative to the control (i.e., lacking test serum).

Because Th2-biased airway inflammation has been associated with VAERD in seronegative children who received FI-RSV and were subsequently exposed to natural RSV infection [18,19,21], secondary objectives were to describe RSVpreF-elicited cell-mediated immune responses at both RSVpreF dose levels (60 µg; 120 µg), within each age stratum (2–<5 year olds; 5–<18 year olds), and within both risk categories of 5–<18 year olds (healthy; high-risk). Cell-mediated immune responses, which included fold rises in RSV F antigen-specific T cells secreting interferon-gamma (IFN-γ; T-helper type 1 (Th1) cytokine) or interleukin-4 (IL-4; Th2 cytokine) from before to 1 month after vaccination, were evaluated using the IFN-γ and IL-4 enzyme-linked immunosorbent spot (ELISpot) assay. The qualified ELISpot assay captures antibodies specific to the cytokines of interest. In the ELISpot assay used in the current analyses, IFN-γ and IL-4 secretion in PBMCs was detected and quantified in polyvinylidene fluoride microplates after antigen-specific stimulation using an RSV F peptide library compared with control (i.e., dimethyl sulfoxide media-unstimulated responses); phytohemagglutinin was used as a positive control. The spot-forming cells (SFC) per well were quantified and reported as SFC per 10^6^ PBMC. The RSV F ELISpot limit of detection values were 20 SFCs per million PBMCs for IFN-γ and 4 SFCs per million PBMCs for IL-4.

Percentages of participants with RSV-A and RSV-B neutralizing titer seroresponses were assessed as an exploratory endpoint. Seroresponse was defined as achieving a ≥4-fold rise from before vaccination if the baseline measurement was above the lower limit of quantitation (LLOQ). If the baseline measurement was below the LLOQ, a postvaccination assay result ≥4 times the LLOQ was considered a seroresponse.

### 2.4. Statistics

Study sample size was not based on statistical criteria. Analyses were conducted using SAS/STAT^®^ (version 9.4, SAS Institute, Cary, NC, USA).

Safety assessments were completed in the safety population (all enrolled participants who received study vaccination as assigned). Immunogenicity assessments were completed in the evaluable immunogenicity population (all eligible participants who received study vaccination as assigned and had the 1-month blood sample collected 27 to 42 days after vaccination, had at least one valid and determinate assay result for the 1 month after vaccination sample, and no major protocol violations).

The two-sided 95% CIs for binary safety variables were constructed using the Clopper-Pearson method. Continuous immunogenicity endpoints were logarithmically transformed for analysis. GMTs and associated two-sided 95% CIs were derived by calculating group means and confidence limits on the natural log scale, based on the Student *t* distribution, and exponentiating the results. GMFRs were calculated as the group mean of the difference of logarithmically transformed assay results (later minus earlier time point) and exponentiating the mean. The associated two-sided 95% CIs were constructed using the Student *t* distribution for the mean difference on the logarithm scale and exponentiating confidence limits. For PBMC analyses, descriptive statistics, including the median and first and third quartiles, were computed. The exact Clopper–Pearson two-sided 95% CI was determined for percentages of participants with seroresponse.

## 3. Results

### 3.1. Participants

**Figure 1:**
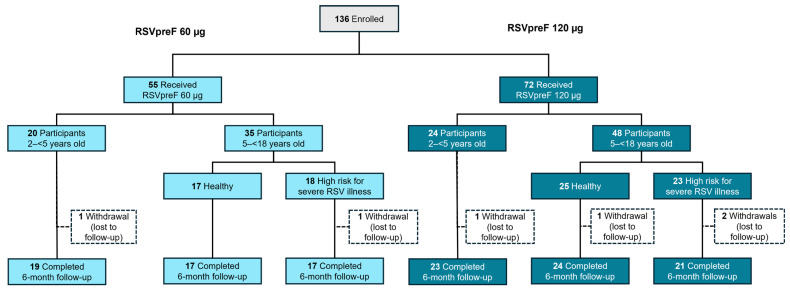
Participant disposition. Of 136 participants enrolled in the study, eight did not pass screening (2–<5 years of age, *n* = 7; 5–<18 years of age, *n* = 1) and one participant was assigned to a vaccine group but did not receive study vaccination. Fourteen participants had important protocol deviations; dosing or administration error was the most common important protocol deviation, occurring in nine participants who were administered RSVpreF 120 µg in error instead of their assigned intervention of RSVpreF 60 µg. Other important protocol deviations were PBMC samples not collected (*n* = 2), serology sample not collected (*n* = 2), whole-blood samples for PBMC processed incorrectly (*n* = 1), incorrect whole-blood sample volume collected for PBMC (*n* = 1); and dose administration into the thigh muscle (*n* = 1). PBMC, peripheral blood mononuclear cells; RSV, respiratory syncytial virus; RSVpreF, bivalent respiratory syncytial virus prefusion F vaccine.

The study enrolled 136 participants. Eight did not pass screening, including three participants 2–<5 years of age who tested RSV seronegative; one participant was assigned to a vaccine group but was not vaccinated. Among the 127 participants receiving RSVpreF, 55 received a 60 µg dose level (2–<5 years of age, *n* = 20; 5–<18 years of age, *n* = 35) and 72 received a 120 µg dose level (*n* = 24 and *n* = 48, respectively); 121 participants completed the 6-month follow-up (Figure 1).

Overall, 54% (69/127) of participants were male, 69% (87/127) were White, and 22% (28/127) were Black (Table 1). Among all participants, 73% (93/127) had reportable medical history, with asthma (31% (40/127)) and seasonal allergy (22% (28/127)) being the most common conditions reported. In 5–<18 year olds with high-risk conditions, asthma was the most common prespecified chronic medical condition, reported by 94% (17/18) and 87% (20/23) of participants who received RSVpreF 60 µg and 120 µg, respectively.

### 3.2. Safety

**Figure 2:**
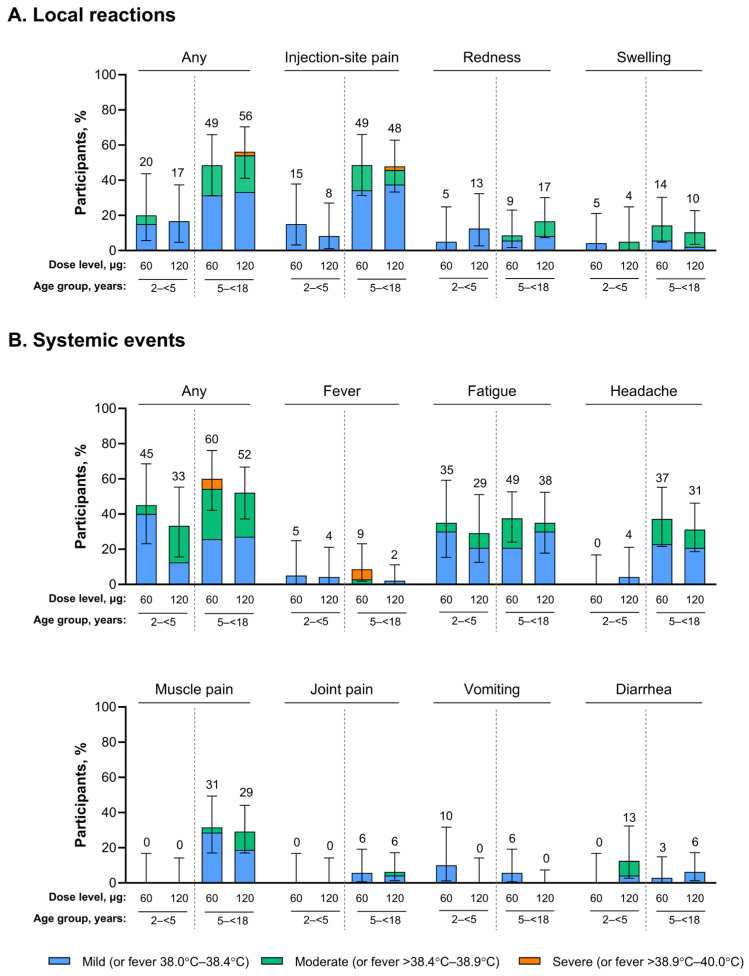
(**A**) Local reactions and (**B**) systemic events within 7 days after vaccination. Data are for the safety population. N = 20 and N = 35 for 2–<5 year olds and 5–<18 year olds, respectively, who received RSVpreF 60 µg; N = 24 and N = 48, respectively, for those receiving RSVpreF 120 µg. Error bars are the 95% CIs. The numbers above the bars are the percentage of participants with the event overall. RSVpreF, bivalent respiratory syncytial virus prefusion F vaccine.

Local reactions within 7 days were reported less frequently among participants 2–<5 years of age than in participants 5–<18 years of age who received RSVpreF 60 µg (20% (4/20) vs. 49% (17/35)) or RSVpreF 120 µg (17% (4/24) vs. 56% (27/48); Figure 2A). Local reactions were generally mild or moderate in severity. The most common local reaction in the younger group was injection-site pain and redness in those receiving RSVpreF 60 µg (15% (3/20) and 5% (1/20)) and RSVpreF 120 µg (8% (2/24) and 13% (3/24)), respectively; the most common local reaction in the older group across both dose levels was injection-site pain (49% (17/35) and 48% (23/48) for 60 µg and 120 µg, respectively). One 5–<18-year-old participant who received RSVpreF 120 µg experienced severe injection-site pain, which resolved in 4 days. Across both age and dose-level groups, the onset of local reactions occurred a median of 1 to 3 days after vaccination and lasted for 1 to 3 days.

Systemic events were also reported less frequently among the younger versus older group for RSVpreF 60 µg (45% (9/20) vs. 60% (21/35)) and RSVpreF 120 µg (33% (8/24) vs. 52% (25/48); Figure 2B). Across dose levels, the most common systemic events reported were fatigue in participants 2–<5 years of age (35% (7/20) and 29% (7/24) for 60 µg and 120 µg, respectively) and fatigue (49% (17/35) and 38% (18/48)), headache (37% (13/35) and 31% (15/48)), and muscle pain (31% (11/35) and 29% (14/48)) in participants 5–<18 years of age. Most systemic events in either age or dose-level group were mild or moderate in severity; severe fever, which resolved in 2 to 3 days, was reported for two participants 5–<18 years of age who received RSVpreF 60 µg. In participants 2–<5 years of age across both dose-level groups, the onset of systemic events occurred a median of 1 to 3 days after vaccination and lasted for 1 to 7 days. Corresponding median onset and duration of systemic events in 5–<18 year olds across both dose-level groups were 1 to 6 and 1 to 2.5 days.

The frequency and severity of reactogenicity events among participants 5–<18 years of age were generally similar in the healthy and high-risk subgroups (Figures S1 and S2). Across RSVpreF dose levels, any local reactions occurring within 7 days after vaccination were reported by 41–60% of the subgroup of healthy 5–<18 year olds and 52–56% in the high-risk subgroup. Across RSVpreF dose levels, any systemic events occurring within 7 days after vaccination were reported by 44–59% of healthy 5–<18 year olds and 61% of high-risk 5–<18 year olds.

**Figure 3:**
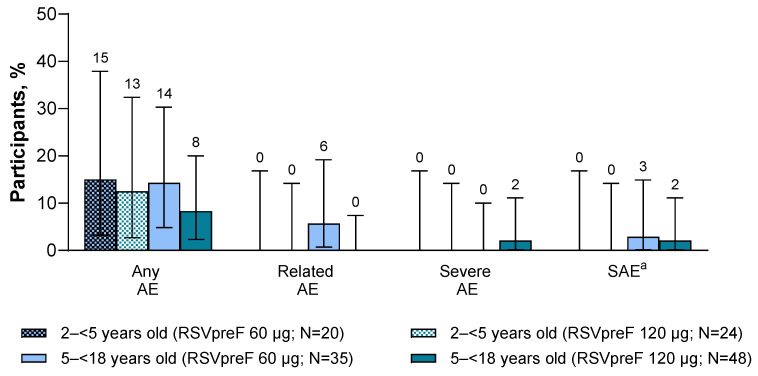
Summary of adverse events. Data are for the safety population and are presented as percentages. Error bars are the 95% CIs. ^a^ Occurring throughout the study. AE, adverse event; RSVpreF, bivalent respiratory syncytial virus prefusion F vaccine; SAE, serious adverse event.

Across age and dose-level groups, reported AEs ranged from 8% (4/48) to 15% (3/20) of participants through 1 month after vaccination (Figure 3). Two participants 5–<18 years of age with a history of asthma had AEs considered by the investigator to be related to RSVpreF 60 µg (axillary pain on Day 1 (mild, resolved after 3 days); abdominal pain on Day 2 (mild, resolved after 1 day)). Through the end of the study, two unrelated SAEs were reported: one in a 5–<18-year-old who received RSVpreF 60 µg (severe asthma occurring on Day 39, which resolved after 11 days, in a participant with history of asthma and atopic dermatitis) and another who received RSVpreF 120 µg (severe nut allergy on Day 1, which resolved after 2 days, in a participant with history of food allergy and asthma). No AEs leading to withdrawal, NDCMCs, or AESIs were reported in either age or dose-level group through the end of the study.

### 3.3. Immunogenicity

**Figure 4:**
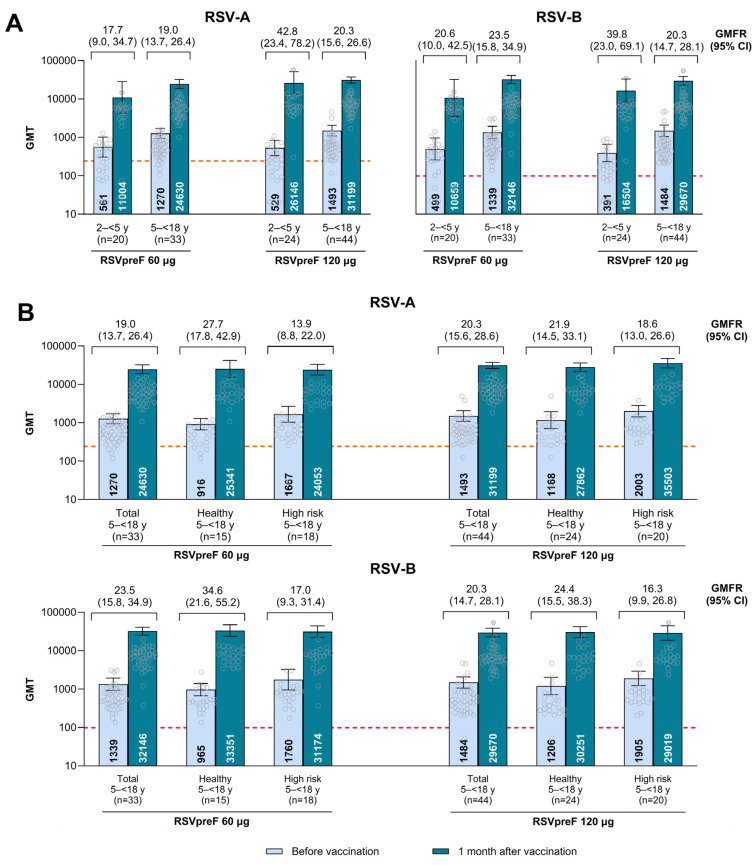
RSV neutralizing GMTs and GMFRs (**A**) overall and (**B**) by risk group of 5–<18 year olds. Data are for the evaluable immunogenicity population. The LLOQ for each neutralization titer was 242 for RSV-A (orange dashed line) and 99 for RSV-B (pink dashed line). Assay results <LLOQ were set to 0.5 × LLOQ for analysis, except for calculating the fold-rise when an assay value before vaccination was <LLOQ but a corresponding assay value after vaccination was ≥LLOQ, where the LLOQ was set for before vaccination. Error bars are the 95% CIs. GMTs and GMFRs and two-sided 95% CIs were calculated by exponentiating the mean logarithm of the titers or of the fold rises, respectively, and the corresponding CIs (based on the Student *t* distribution). GMT, geometric mean titer; GMFR, geometric mean fold rise; LLOQ, lower limit of quantitation; RSV, respiratory syncytial virus; RSVpreF, bivalent respiratory syncytial virus prefusion F vaccine.

One month after vaccination, RSVpreF elicited robust immune responses in both age groups and dose levels (Figure 4A). RSV-A and RSV-B serum neutralizing GMTs across dose levels were lower in participants 2–<5 years of age than in participants 5–<18 years of age both at baseline (391–561 vs. 1270–1493) and 1 month after vaccination (10,659–26,146 vs. 24,630–32,146); similar or higher GMFRs were observed in the younger versus the older group (17.7–42.8 vs. 19.0–23.5). In participants 2–<5 years of age, GMTs and GMFRs were lower in response to RSVpreF 60 µg at 1 month after vaccination compared with RSVpreF 120 µg (RSV-A and RSV-B GMTs of 11,004 and 10,659 vs. 26,146 and 16,504; RSV-A and RSV-B GMFRs of 17.7 and 20.6 vs. 42.8 and 39.8). Responses to both RSVpreF 60 µg and 120 µg dose levels were comparable in participants 5–<18 years of age (RSV-A and RSV-B GMTs of 24,630 and 32,146 vs. 31,199 and 29,670; RSV-A and RSV-B GMFRs of 19.0 and 23.5 vs. 20.3 and 20.3). Observed GMFRs for both RSV-A and RSV-B were generally similar among age groups and dose levels.

RSVpreF responses at 1 month after vaccination were similarly robust between healthy 5–<18 year olds and those with high-risk conditions (Figure 4B). At baseline, RSV-A and RSV-B serum neutralizing GMTs across dose levels were 916–1206 in the subgroup of healthy participants 5–<18 years of age and 1667–2003 in the high-risk subgroup. Corresponding values were similar between these healthy and high-risk subgroups at 1 month after vaccination (25,341–33,351 vs. 24,053–35,503); higher GMFRs were observed in the healthy versus high-risk subgroup of 5–<18 year olds (21.9–34.6 vs. 13.9–18.6).

Percentages of participants with seroresponse followed similar patterns as for GMTs and GMFRs (Figure S3). One month after vaccination of participants 2–<5 years of age, the percentage of participants achieving seroresponse against RSV-A and RSV-B were both 85% for RSVpreF 60 µg and 92% and 96%, respectively, for RSVpreF 120 µg. One month after vaccination of participants 5–<18 years of age, the percentage of participants achieving seroresponse against RSV-A and RSV-B was ≥93% for both RSVpreF dose levels.

Six participants 2–<5 years of age who were RSV-seropositive had both RSV-A and RSV-B neutralizing titers less than the LLOQ at baseline; five of these children (60 µg, *n* = 3; 120 µg, *n* = 2) showed poor to no serologic response after vaccination (i.e., neutralizing titers ≤ 721 and ≤69 for RSV-A and RSV-B, respectively; Figure S4). There were no 5–<18-year-old participants who had both RSV-A and RSV-B baseline titers < LLOQ.

A post hoc analysis suggested that 2–<5 year olds with the lowest baseline RSV neutralizing titers (i.e., first quartile across the range of baseline values) had lower neutralizing titers 1 month after vaccination for both RSVpreF 60 µg and 120 µg than those with higher baseline neutralizing titers (i.e., second through fourth quartiles across the range of baseline values; Figure S5).

Analysis of T-cell responses from PBMCs at baseline and 1 month after vaccination showed that median IFN-γ fold rises 1 month after vaccination in 2–<5 year olds (2.2 and 2.1 for RSVpreF 60 µg and 120 µg, respectively) were slightly lower than those observed in 5–<18 year olds (3.2 and 4.8; Figure S6). No notable IL-4 increases were observed in either age group at either dose level at 1 month after vaccination, suggesting a Th1-dominant CD4+ T-cell response.

## 4. Discussion

Although RSV-LRTI occurs at a high rate in infancy, it also causes serious medically attended illness throughout the first 5 years of life as well as in pediatric populations with high-risk conditions [1,2,3,4,5]. Clinical management of severe RSV-LRTI is based largely on supportive care and prevention (via direct immunization, protection through maternal immunization, or directly administered antibodies, which is not available for children beyond the second year of life) [4,5,6,7,11,13,14,17,34,35,36,37]. Therefore, the availability of an RSV vaccine for direct immunization for the older at-risk pediatric population remains an unmet need.

In RSV-seropositive children and adolescents, we found that a single RSVpreF vaccination at either the 60 µg or 120-µg dose level was well tolerated with an acceptable safety profile. Reactogenicity events at both RSVpreF dose levels occurred at similar frequences, and these events, particularly local reactions, were more common in older children and adolescents 5–<18 years of age than in children 2–<5 years of age; most events were mild to moderate in severity. Age-group-related differences in the frequency and severity of specific reactogenicity events are expected and have been observed with other vaccines used for this age group, including pneumococcal vaccines [38,39].

In RSV-seropositive children and adolescents, a single RSVpreF vaccination at the 60 µg and 120 µg dose levels elicited robust neutralizing and Th1-biased T-cell responses 1 month after vaccination. Responses to RSVpreF 120 µg in both age groups were similar to or greater than those of adults in whom efficacy was demonstrated [28]. Additionally, responses in pediatric participants with chronic conditions were consistent with those observed in phase 3 trials in adult populations. Neutralizing responses were also balanced between RSV-A and RSV-B, which may be important for protection against all RSV disease regardless of RSV subgroup because both disease-causing subgroups circulate variably and cause severe disease in children [28,30,40,41].

For the older age group of children and adolescents, further investigation of a single dose of RSVpreF 120 µg in later-phase trials is supported by data from the present study and would be the same RSVpreF dose level licensed for adults (during pregnancy; in 18–59 year olds with increased risk for RSV-LRTI; and in those ≥60 years of age regardless of underlying RSV-LRTI risk) [12]. Notably, the 120 µg dose level in 2–<5-year-old children elicited higher neutralizing antibody responses than the 60 µg dose level. RSVpreF 120 µg may provide improved protective immunity in this age group; however, in the absence of an established correlate or threshold of protection, this remains unclear. We expect that active immunization with RSVpreF in an older pediatric population will provide similar protection to that in adults. Future trials are planned and will continue to investigate the relationship between RSV neutralizing titers and efficacy in all age groups including pediatric populations. Post hoc analyses of baseline neutralizing titers in 2–<5 year olds found those with the lowest baseline titers (i.e., in the first quartile) had the highest fold-rise but overall lower postvaccination neutralizing titers than those with higher baseline titers (i.e., in the second through fourth quartiles). Reasons for this variability in baseline neutralizing titers among this younger group have not been elucidated, but analyses in older populations have suggested an association between low baseline RSV neutralizing titers and timing of previous RSV infection and demographic characteristics (e.g., living in larger households or those with young children; attending daycare) [42,43]. Our post hoc analyses suggest the 2–<5 years of age group may require a higher RSVpreF vaccine dose level or a multidose schedule. The approved RSVpreF 120 µg dose level in adults has demonstrated prolonged protection, suggesting efficacy even with waning neutralizing titers [28]. Nonetheless, additional dose-finding studies may help to ascertain an optimal RSVpreF dose level and/or schedule in this younger group [44]. Need for a multidose series would be consistent with some early childhood vaccines that require more than one dose to mount durable immune responses [45].

One month after RSVpreF vaccination, increases in RSV F-specific, IFN-γ-secreting T-cell responses, with negligible increases in IL-4-secreting T-cell responses, were observed in both age groups and with both dose levels. This Th1-dominant response is consistent with responses observed in adults and contrasts to the Th2-dominant response elicited by the FI-RSV vaccine in RSV-seronegative infants that was associated with VAERD in clinical trials performed in the 1960s [18,46]. However, because this was a descriptive post hoc analysis, these findings from our trial should be interpreted with caution.

Our study predated the July 2024 hold that the US Center for Biologics Evaluation and Research placed on clinical study enrollment of RSV vaccine candidates in <2-year-old children and RSV-naive 2–5 year olds after imbalances in severe RSV cases were identified in a phase 1 trial in which infants received RSV/human metapneumovirus-modified mRNA vaccine candidates (mRNA-1345, mRNA-1365) at 5–<8 months of age, but not in children vaccinated at 8–<24 months of age (about half of whom were baseline RSV seronegative) [47]. The US Food and Drug Administration subsequently held a Vaccines and Related Biological Products Advisory Committee meeting in December 2024 to discuss these results and continue to maintain the clinical hold for non-live attenuated RSV vaccines in children younger than 5 years, including for clinical investigation of direct immunization with RSVpreF in this age group.

Study limitations include that this was a phase 1 study conducted in the United States in a limited population; therefore, results are descriptive and may not be generalizable to a larger and more diverse population. Additionally, the study was limited to seropositive children; thus, RSVpreF safety in RSV-seronegative children remains unknown. Safety and immunogenicity follow-up in our study was also limited to 6 months after vaccination and systematic surveillance for RSV-LRTI was not performed; however, SAEs (including any hospitalization) were collected throughout the study. Other limitations include the open-label, uncontrolled study design. Lastly, subgroups based on age alone did not consider other factors such as body weight; however, baseline serostatus and neutralizing titer levels, rather than other factors, appear to be the most important driver of immune response to RSVpreF.

## 5. Conclusions

RSVpreF was safe and well tolerated and elicited serum neutralizing and Th1-dominant cell-mediated immune responses in RSV-seropositive participants 2–<18 years of age at both the 60 µg and 120 µg dose levels. These results support further clinical development of RSVpreF in a seropositive pediatric population, including those with chronic conditions. Importantly, future studies of younger children who are RSV-seronegative will need to take into consideration current and developing understandings of potential mechanisms of RSV VAERD.

## Figures and Tables

**Table 1:** Demographic and baseline clinical characteristics.

Characteristic	RSVpreF 60 µg				RSVpreF 120 µg				Overall Total (N = 127)
	2–<5 Years Old (N = 20)	5–<18 Years Old			2–<5 Years Old (N = 24)	5–<18 Years Old			
		Healthy (N = 17)	High Risk (N = 18)	Total (N = 35)		Healthy (N = 25)	High Risk (N = 23)	Total (N = 48)	
Sex, *n* (%)									
Male	13 (65)	12 (71)	11 (61)	23 (66)	11 (46)	10 (40)	12 (52)	22 (46)	69 (54.3)
Female	7 (35)	5 (29)	7 (39)	12 (34)	13 (54)	15 (60)	11 (48)	26 (54)	58 (45.7)
Race, *n* (%)									
White	15 (75)	13 (77)	9 (50)	22 (63)	17 (71)	16 (64)	17 (74)	33 (69)	87 (68.5)
Black	4 (20)	2 (12)	6 (33)	8 (23)	3 (13)	7 (28)	6 (26)	13 (27)	28 (22.0)
Asian	0	0	0	0	0	2 (8)	0	2 (4)	2 (1.6)
American Indian or Alaska Native	0	0	0	0	1 (4)	0	0	0	1 (0.8)
Multiracial	1 (5)	2 (12)	3 (17)	5 (14)	3 (13)	0	0	0	9 (7.1)
Ethnicity, *n* (%)									
Hispanic/Latino	5 (25)	3 (18)	4 (22)	7 (20)	1 (4)	5 (20)	0	5 (10)	18 (14.2)
Non-Hispanic/non-Latino	15 (75)	14 (82)	13 (72)	27 (77)	22 (92)	20 (80)	23 (100)	43 (90)	107 (84.3)
Not reported	0	0	1 (6)	1 (3)	1 (4)	0	0	0	2 (1.6)
Age at vaccination, years									
Mean (SD)	3.2 (0.8)	9.5 (3.6)	9.6 (3.4)	9.6 (3.4)	3.0 (0.8)	12.6 (4.6)	11.0 (3.5)	11.8 (4.1)	8.2 (4.9)
Any prespecified medical history, *n* (%) ^a^	1 (5)	0	18 (100)	18 (51)	3 (13)	0	23 (100)	23 (48)	45 (35.4)
Cystic fibrosis	0	0	1 (6)	1 (3)	0	0	0	0	1 (0.8)
Asthma	1 (5)	0	17 (94)	17 (49)	2 (8)	0	20 (87)	20 (42)	40 (31.5)
Pulmonary malformation	0	0	0	0	0	0	1 (4)	1 (2)	1 (0.8)
Down syndrome	0	0	1 (6)	1 (3)	1 (4)	0	1 (4)	1 (2)	3 (2.4)
Cerebral palsy	0	0	0	0	0	0	2 (9)	2 (4)	2 (1.6)

Data are for the safety population. RSVpreF, bivalent respiratory syncytial virus prefusion F vaccine. ^a^ No participant had chronic respiratory disease, neuromuscular disease, or congenital heart disease.

## Data Availability

Upon request, and subject to review, Pfizer will provide the data that support the findings of this study. Subject to certain criteria, conditions, and exceptions, Pfizer may also provide access to the related individual de-identified participant data. See https://www.pfizer.com/science/clinical-trials/trial-data-and-results (accessed 18 January 2026) for more information.

## Supplementary Materials

The following supporting information can be downloaded at: https://www.mdpi.com/article/10.3390/vaccines14020128/s1, Table S1. Grading scale for local reactions and systemic events. Figure S1. Local reactions within 7 days after vaccination by risk category. Figure S2. Systemic events within 7 days after vaccination by risk category. Figure S3. Percentage of participants with RSV-A and RSV-B neutralizing titer seroresponse 1 month after vaccination. Figure S4. Scatter plot of neutralizing GMTs before versus 1 month after receipt of RSVpreF. Figure S5. Neutralizing GMTs 1 month after receipt of RSVpreF by baseline quartile GMTs in 2–<5-year-olds. Figure S6. RSV F antigen−specific T cells expressing IFN-γ and IL-4 fold rise.
