# Supplementary material for: Bivalent RSVpreF Subunit Vaccine Safety and Immunogenicity in Seropositive 2–<18 Year Olds
Source: Vaccines (Basel). 2026 Jan 28;14(2):128. doi: 10.3390/vaccines14020128 (PMC12944973; doi:10.3390/vaccines14020128)

Figure S2. Systemic events within 7 days after vaccination by risk category

Data are for the safety population of 5–<18-year-olds. **Panel A** shows systemic events in RSVpreF 60-µg recipients and **Panel B** shows systemic events in RSVpreF 120-µg recipients. N values for RSVpreF 60-µg recipients are 35, 17, and 18 for the total, healthy, and high-risk groups, respectively. Corresponding N values for RSVpreF 120-µg recipients are 48, 25, and 23. Error bars are the 95% CIs. RSVpreF, bivalent respiratory syncytial virus prefusion F vaccine.

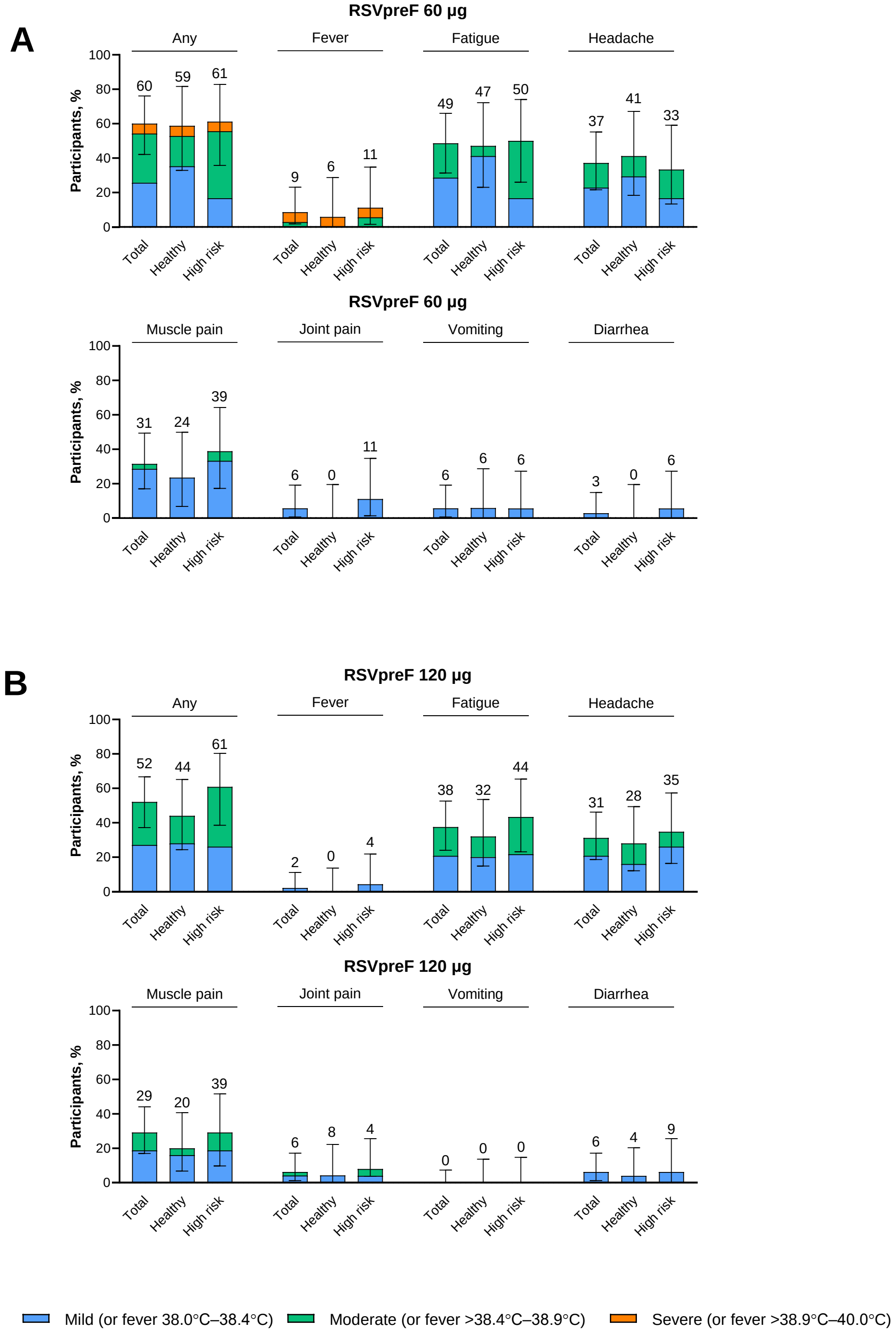

Supplement: Supplementary file 1 [file vaccines-14-00128-s001.zip › vaccines-4062096_Figure S2.pdf]
