# Supplementary material for: Bivalent RSVpreF Subunit Vaccine Safety and Immunogenicity in Seropositive 2–<18 Year Olds
Source: Vaccines (Basel). 2026 Jan 28;14(2):128. doi: 10.3390/vaccines14020128 (PMC12944973; doi:10.3390/vaccines14020128)

Figure S3. Percentage of participants with RSV-A and RSV-B neutralizing titer seroresponse 1 month after vaccination

Data are for the evaluable immunogenicity population. The LLOQ for each neutralization titer was 242 for RSV-A and 99 for RSV-B. Seroresponse was defined as achieving a ≥4-fold rise from before vaccination, if the baseline measurement was above the LLOQ. If the baseline measurement was below the LLOQ, a postvaccination assay result ≥4 × LLOQ was considered a seroresponse. Error bars are the 95% CIs determined by the Clopper-Pearson method. LLOQ, lower limit of quantitation; RSV, respiratory syncytial virus; RSVpreF, bivalent respiratory syncytial virus prefusion F vaccine.

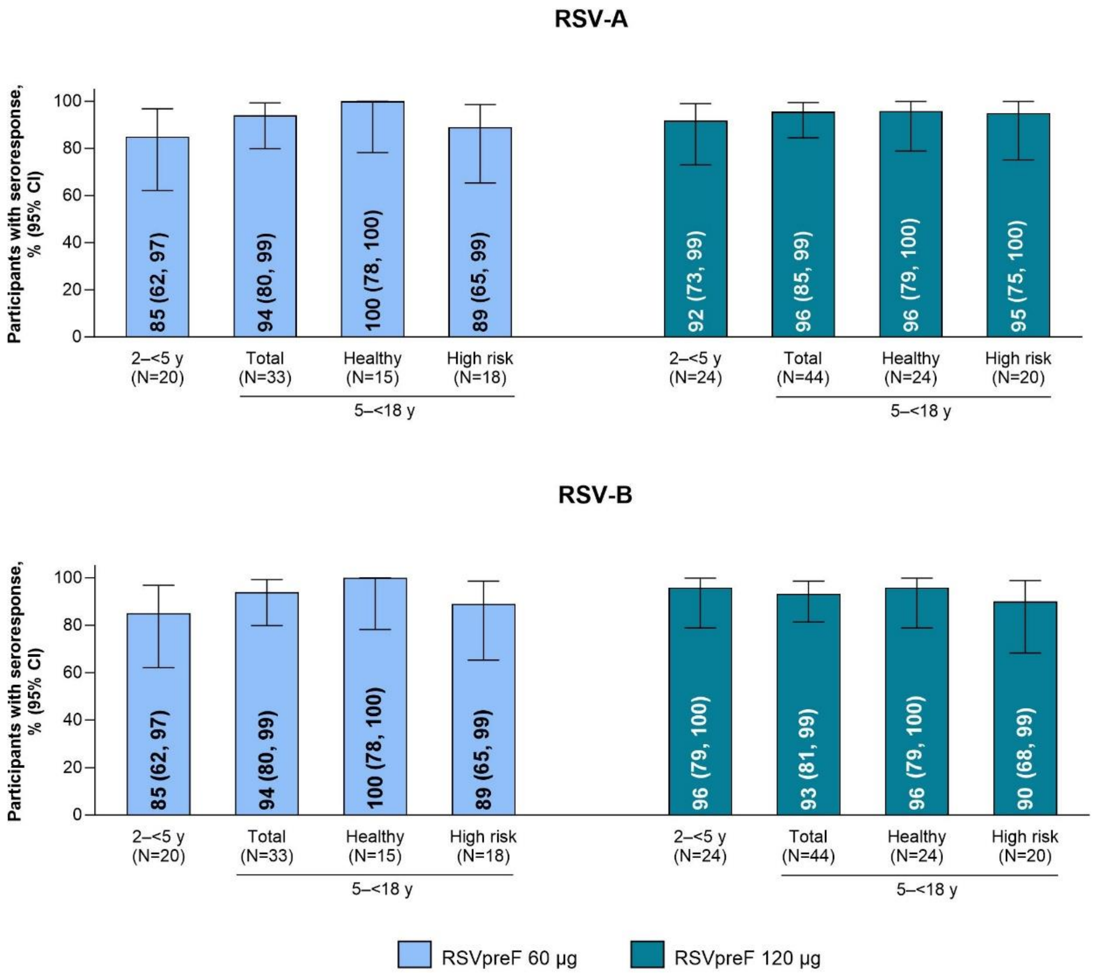

Supplement: Supplementary file 1 [file vaccines-14-00128-s001.zip › vaccines-4062096_Figure S3.pdf]
