# Supplementary material for: Bivalent RSVpreF Subunit Vaccine Safety and Immunogenicity in Seropositive 2–<18 Year Olds
Source: Vaccines (Basel). 2026 Jan 28;14(2):128. doi: 10.3390/vaccines14020128 (PMC12944973; doi:10.3390/vaccines14020128)

Figure S4. Scatter plot of neutralizing GMTs before versus 1 month after receipt of RSVpreF

Data are for the randomized population. The LLOQ for each neutralization titer was 242 for RSV-A (orange dashed line) and 99 for RSV-B (pink dashed line). Assay results <LLOQ were set to 0.5 × LLOQ for analysis, except for calculating the fold-rise when an assay value before vaccination was <LLOQ but a corresponding assay value after vaccination was ≥LLOQ, where the LLOQ was set for before vaccination. GMT, geometric mean titer; LLOQ, lower limit of quantitation; RSV, respiratory syncytial virus; RSVpreF, bivalent respiratory syncytial virus prefusion F vaccine.

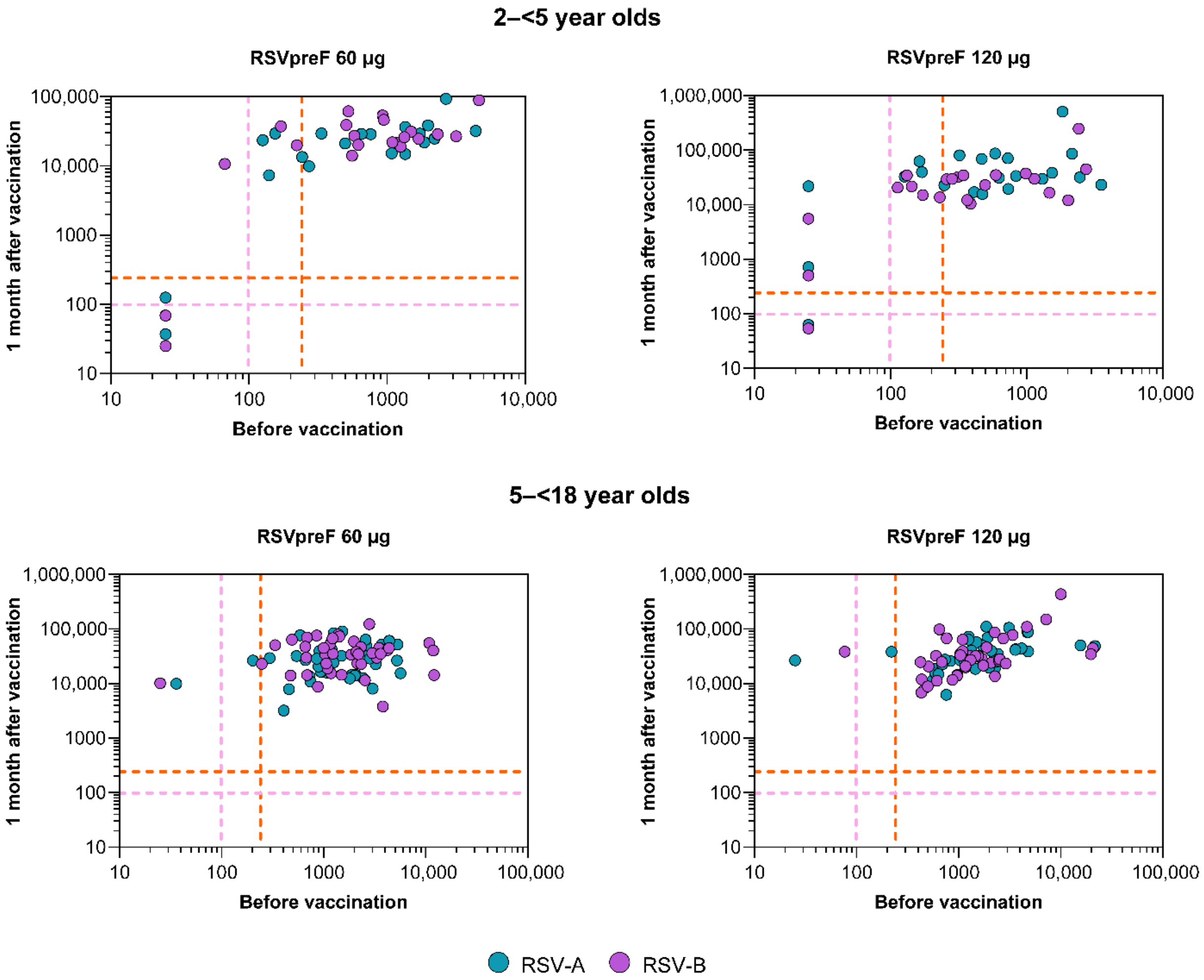

Supplement: Supplementary file 1 [file vaccines-14-00128-s001.zip › vaccines-4062096_Figure S4.pdf]
