# Supplementary material for: Bivalent RSVpreF Subunit Vaccine Safety and Immunogenicity in Seropositive 2–<18 Year Olds
Source: Vaccines (Basel). 2026 Jan 28;14(2):128. doi: 10.3390/vaccines14020128 (PMC12944973; doi:10.3390/vaccines14020128)

Figure S5. Neutralizing GMTs 1 month after receipt of RSVpreF by baseline quartile GMTs in 2–<5-year-olds

Data are for the evaluable immunogenicity population. **Panel A** shows results for RSVpreF 60-µg recipients and **Panel B** shows results for RSVpreF 120-µg recipients. The forest plots show the GMR (GMT for Q1 to GMT for Q2–Q4) at 1 month after vaccination. Error bars are the 95% CIs. The range of neutralizing GMTs at baseline for the Q1 group were 25–148 and 25–197 for RSV-A and RSV-B, respectively, in those who received RSVpreF 60 µg and 25–210 and 25–158 in those who received RSVpreF 120 µg. The range of neutralizing GMTs at baseline for the Q2–Q4 group were 148–4395 and 197–4624 for RSV-A and RSV-B in those who received RSVpreF 60 µg and 210–3535 and 158–2734 in those who received RSVpreF 120 µg. GMR, geometric mean ratio; GMT, geometric mean titer; Q, quartile; RSV, respiratory syncytial virus; RSVpreF, bivalent respiratory syncytial virus prefusion F vaccine.

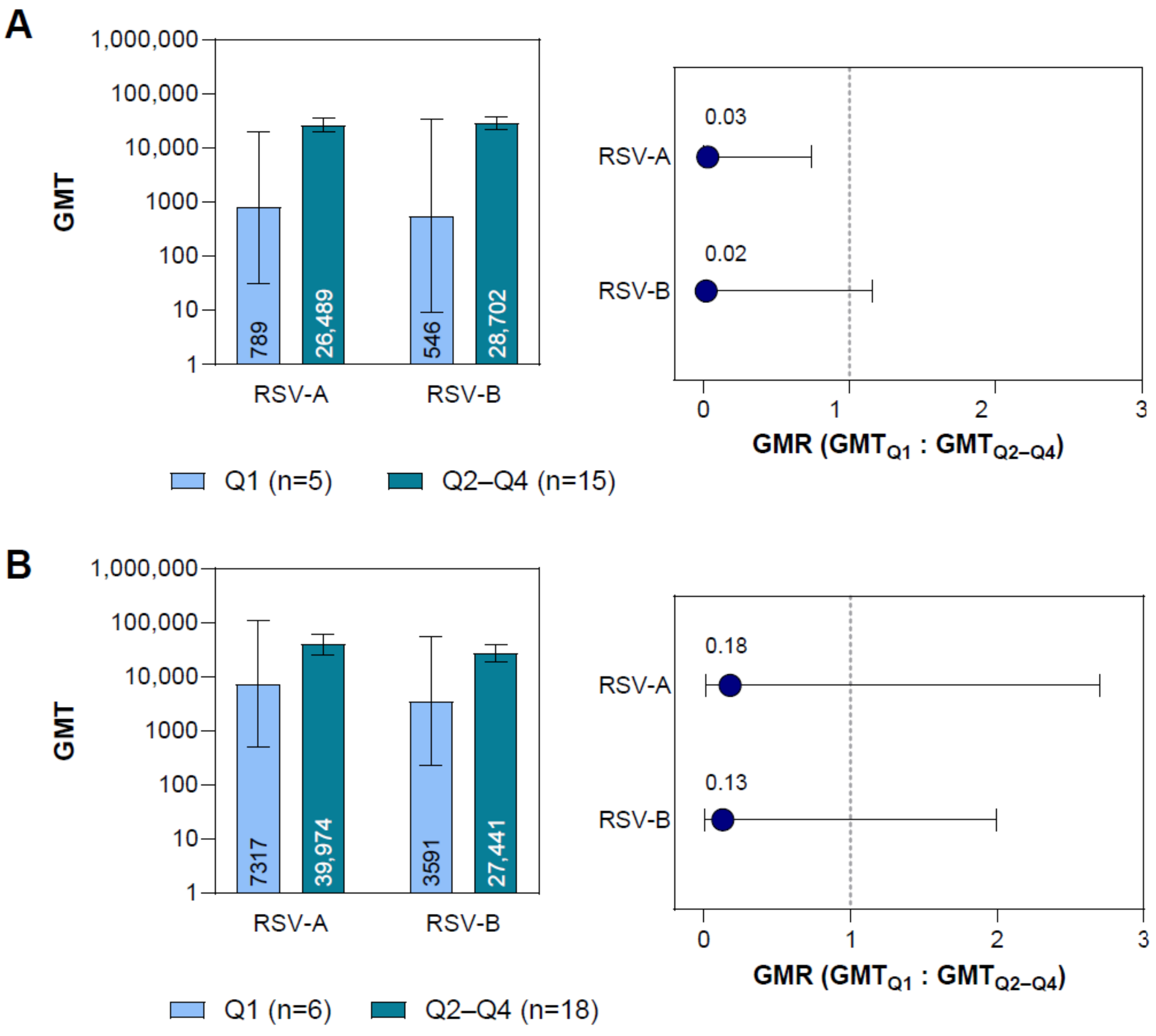

Supplement: Supplementary file 1 [file vaccines-14-00128-s001.zip › vaccines-4062096_Figure S5.pdf]
