# Supplementary material for: Bivalent RSVpreF Subunit Vaccine Safety and Immunogenicity in Seropositive 2–<18 Year Olds
Source: Vaccines (Basel). 2026 Jan 28;14(2):128. doi: 10.3390/vaccines14020128 (PMC12944973; doi:10.3390/vaccines14020128)

Figure S6. RSV F antigen–specific T cells expressing IFN-γ and IL-4 fold rise

Data are for the evaluable immunogenicity population. The fold rise is from before vaccination to 1 month after vaccination. The RSV F ELISpot LOD values were as follows: IFN-γ=20 SFC/million PBMCs; IL-4=4 SFC/million PBMCs. Assay results <LOD were set to 0.5 × LOD for analysis, except for calculating the fold rise when an assay value before vaccination was <LOD but a corresponding assay value after vaccination was ≥LOD, where the LOD was set for before vaccination. ELISpot, enzyme-linked immune absorbent spot assay; F, F-specific peptides; IFN-γ, interferon gamma; IL-4, interleukin 4; LOD, limit of detection; PBMC, peripheral blood mononuclear cell; Q1, first quartile; Q3, third quartile; RSV, respiratory syncytial virus; RSVpreF, bivalent respiratory syncytial virus prefusion F vaccine; SFC, spot-forming cell.

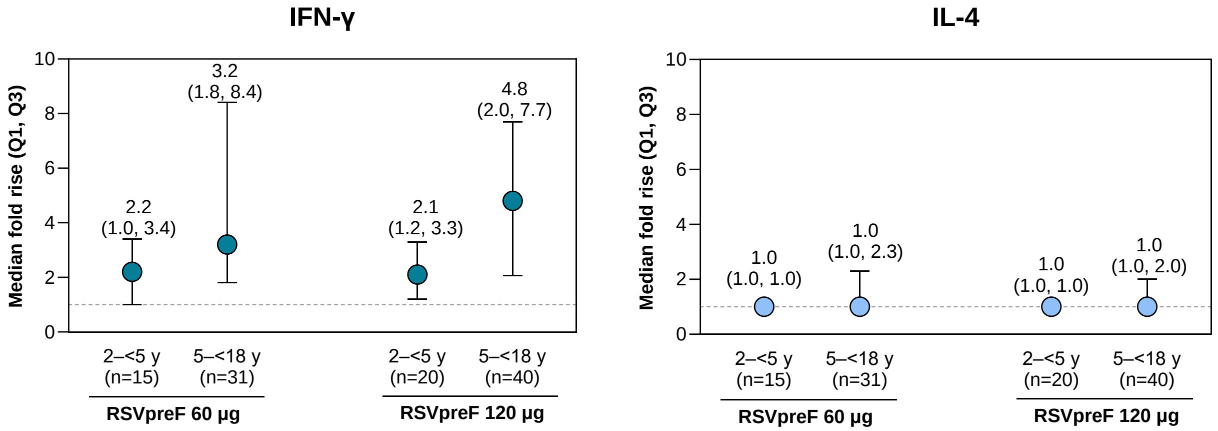

Supplement: Supplementary file 1 [file vaccines-14-00128-s001.zip › vaccines-4062096_Figure S6.pdf]
