# Supplementary material for: Bivalent RSVpreF Subunit Vaccine Safety and Immunogenicity in Seropositive 2–<18 Year Olds
Source: Vaccines (Basel). 2026 Jan 28;14(2):128. doi: 10.3390/vaccines14020128 (PMC12944973; doi:10.3390/vaccines14020128)
Supplement: Supplementary file 1 [file vaccines-14-00128-s001.zip › vaccines-4062096_Supplementary Text.pdf]

SUPPLEMENTARY TEXT

Participant Recruitment

US study sites with pediatric vaccine clinical trial experience were selected. Participant recruitment was primarily based on pediatric patient database with pediatric/family physician referrals.

Eligibility Criteria

Participants were included if all the following criteria applied:

- 2–<18 years of age at enrollment.
- Either healthy or considered by the investigator to be at high risk of respiratory syncytial virus (RSV) disease based on the presence of one of the following chronic medical conditions:
  - Cystic fibrosis
  - Medically treated asthma
  - Other chronic respiratory diseases and malformations of the lung
  - Down syndrome
  - Neuromuscular disease
  - Cerebral palsy
  - Hemodynamically significant or symptomatic congenital heart disease

Other inclusion criteria included:

- All participants 2–<5 years of age were to be seropositive for RSV as confirmed by serology.
- Participants' parent(s)/legal guardian(s) and participants, as age appropriate, who were willing and able to comply with all scheduled visits, investigational plan, laboratory tests, and other study procedures. Participants' parent(s)/legal guardian(s) were to be available for telephone contact with site staff for the duration of the study.
- The participant's parent(s)/legal guardian could give signed informed consent, which included compliance with the requirements and restrictions listed in the informed consent document and the study protocol. Depending on the age of the participant and according to local requirements, participants were also asked to provide assent as appropriate (verbal or written).

Participants were excluded if any of the following criteria applied:

Medical Conditions

- Immunocompromised individuals associated with known or suspected immunodeficiency, as determined by history or laboratory/physical examination.
- Individuals with a history of autoimmune disease or an active autoimmune disease requiring therapeutic intervention, including but not limited to systemic lupus erythematosus (stable type 1 diabetes and hypothyroidism were permitted).
- Other medical or psychiatric condition, including recent (within the past year) or active suicidal ideation/behavior or laboratory abnormality that may increase the risk of study participation or, in the investigator's judgment, make the participant inappropriate for the study.
- History of severe adverse reaction associated with a vaccine or severe allergic reaction (eg, anaphylaxis) to any component of the study intervention(s).
- Bleeding diathesis or condition associated with prolonged bleeding that would, in the opinion of the investigator, contraindicate intramuscular injection.
- Individuals with a history of epilepsy or other seizure disorders or a history of seizures or other neurologic complications following vaccination.

Previous/Concomitant Therapy

- Previous vaccination with any licensed or investigational RSV vaccine or planned receipt during study participation. Children who may have been exposed to investigational RSV vaccines through maternal immunization were permitted.
- Receipt of investigational or approved monoclonal antibodies against RSV within 6 months before study intervention administration or planned receipt throughout the study.
- Receipt of blood/plasma products or immunoglobulins within 28 days before study intervention administration or planned receipt throughout the study.
- Receipt of chronic systemic treatment with known immunosuppressant medications (including cytotoxic agents or systemic corticosteroids) or radiotherapy within 60 days before study intervention administration or planned receipt throughout the study. Systemic corticosteroids were defined as those administered for ≥14 days at a dose of ≥20 mg/day of prednisone or equivalent (eg, for cancer or an autoimmune disease). Inhaled/nebulized, intra-articular, intrabursal, or topical (skin, eyes, or ears) corticosteroids were permitted.

Previous/Concurrent Clinical Study Experience

- Participation in other studies involving study intervention within 28 days before study entry or for the duration of study participation.

Serostatus Testing

Participant enrollment was based on the precondition of RSV-positive serology determined by an immunochromatographic test (ICT) that detects antibodies to RSV M and/or N in whole blood. The RSV ICT was developed using the ULFA Test Kit (Abcam, Cambridge, UK) and gold-conjugated RSV M and N proteins (Pfizer, Pearl River, NY). The nitrocellulose membrane includes a control line (streptavidin) and a test line (anti-ULFA tag). The liquid reaction mixture contains ULFA-tagged goat anti-human immunoglobulin (Ig) antibodies (Southern Biotech, Birmingham, AL) and gold-conjugated biotin. Upon mixing with a blood sample, human antibodies bind to the conjugated anti-human Ig. A positive test line indicates presence of RSV M- and/or N-specific antibodies in the sample.

List of Study Investigators and Study Sites

Principal Investigator	Sub-Investigator	Location
Boppana, Suresh	Lopez, Michael<br>Pinninti, Swetha<br>Turner, Kenneth	University of Alabama at Birmingham–School of Medicine, Birmingham, AL
Caviness, Alison	Crook, Gretchen*<br>Jain, Sangeeta*<br>Medrano, William*<br>Valla, Noreen*	ARC Clinical Research at Four Points, Austin, TX
Davis, Matthew<br>Casey, Janet*	Foley, Jennifer<br>Glod, Cassidy<br>Hickin, Kristine<br>Kelly, Jean<br>Mooney, Julie<br>Schirmer, Leigh<br>Taddeo, Sarah<br>Davis, Matthew*<br>Davis, Roderick*<br>DiPoala, Joseph*<br>Hallinan, Cherie*	Rochester Clinical Research, LLC, Rochester, NY
Englund, Janet	Korkowski, Sarah<br>Mohan, Kathleen<br>Ruedebusch, Paula	Seattle Children's–Building Cure, Seattle, WA, USA

	Vora, Surabhi<br>Waghmare, Alpana	
Ensz, David	Bechtold, Nicholas*<br>Harrison, Tavane*<br>Loepp, Jenny*	Velocity Clinical Research, Sioux City, Sioux City, IA
Lucksinger, Gregg	Zimmerman, Michael<br>Spalding, Therese*	Velocity Clinical Research, Austin, Austin, TX
Maldonado, Yvonne	Zhang, Hongqing<br>Govindarajan, Prasanthi*	Stanford University Medical Center, Palo Alto, CA
Ohnmacht, Richard	Dayalkumar, Nha Vi<br>Haughey, Lynne<br>Stanton, Ariana	Velocity Clinical Research, Providence, East Greenwich, RI
Paulsen, Grant	Brady, Rebecca*<br>Dickey, Michelle*<br>Frenck, Robert*<br>Kidd, Jamie*<br>Scaggs-Huang, Felicia*<br>Widdice, Eleanor*	Cincinnati Children`s Hospital Medical Center, Cincinnati, OH
Raiser, Frederick	Bauman, Azra<br>Essink, Brandon<br>Gray, Roni<br>Maddox, Stephanie<br>Short, Colleen<br>Wilson, Alan<br>Yovogan, Akossiwa	Velocity Clinical Research, Omaha, Omaha, NE
Richards, John	King, Bari<br>Luth, Lori<br>Rizzardi, Barbara<br>Abad, Vanesa*	Velocity Clinical Research, Salt Lake City, West Jordan, UT
Roque Guerrero, Lilia	Gil Ruiz, Dael<br>Gomez Ramirez, Ana	Bio-Medical Research LLC, Miami, FL
Senders, Shelly	Brennan, Molly*<br>Fillioe, Caitlin *	Senders Pediatrics, South Euclid, OH

	Jezerinac, Nicholas*	
Sher, Lawrence	Palanjian, Alissa	Peninsula Research Associates, Rolling Hills Estates, CA
Smith, Michael	Blatt, Adam<br>Cassas, Christy<br>Walter, Emmanuel	Duke Vaccine and Trials Unit, Durham, NC
Striplin, Scott<br>Jeanfreau, Robert*	Mollasalehi, Jasmine<br>Murray, Joseph<br>Pati, Paul*<br>Pitts, Matthew*	Velocity Clinical Research, Metairie, Metairie, LA

*Previous investigator or sub-investigator.
