# Supplementary material for: Bivalent RSVpreF Subunit Vaccine Safety and Immunogenicity in Seropositive 2–<18 Year Olds
Source: Vaccines (Basel). 2026 Jan 28;14(2):128. doi: 10.3390/vaccines14020128 (PMC12944973; doi:10.3390/vaccines14020128)
Supplement: Supplementary file 1 [file vaccines-14-00128-s001.zip › vaccines-4062096_Table S1.pdf]

Table S1. Grading scale for local reactions and systemic events

	Age group, years	Mild	Moderate	Severe
Local reaction				
Injection-site pain	≥2	Does not interfere with activity	Interferes with activity	Prevents daily activity
Redness	2–<12	1–4 caliper units[a] (0.5–2.0 cm)	5–14 caliper units[a] (>2.0–7.0 cm)	>14 caliper units[a] (>7 cm)
	≥12	5–10 caliper units[a] (>2.0–5.0 cm)	11–20 caliper units[a] (>5.0–10.0 cm)	>20 caliper units[a] (>10 cm)
Swelling	2–<12	1–4 caliper units[a] (0.5–2.0 cm)	5–14 caliper units[a] (>2.0–7.0 cm)	>14 caliper units[a] (>7 cm)
	≥12	5–10 caliper units[a] (>2.0–5.0 cm)	11–20 caliper units[a] (>5.0–10.0 cm)	>20 caliper units[a] (>10 cm)
Systemic event				
Vomiting	≥2	1–2 times in 24 hours	>2 times in 24 hours	Requires intravenous hydration
Diarrhea	≥2	2–3 loose stools in 24 hours	4–5 loose stools in 24 hours	≥6 loose stools in 24 hours
Headache	≥2	Does not interfere with activity	Some interference with activity	Prevents daily routine activity
Fatigue/tiredness	≥2	Does not interfere with activity	Some interference with activity	Prevents daily routine activity
Muscle pain	≥2	Does not interfere with activity	Some interference with activity	Prevents daily routine activity
Joint pain	≥2	Does not interfere with activity	Some interference with activity	Prevents daily routine activity

[a]Or measuring device units.
